# Institutional Delivery Service Utilization among Women from Rural Districts of Wolaita and Dawro Zones, Southern Ethiopia; a Community Based Cross-Sectional Study

**DOI:** 10.1371/journal.pone.0151082

**Published:** 2016-03-17

**Authors:** Mihiretu Alemayehu Arba, Tadele Dana Darebo, Mengistu Meskele Koyira

**Affiliations:** School of Public Health, College of Health Sciences and Medicine, Wolaita Sodo University, Wolaita Sodo, PO Box—138, Ethiopia; Universiti Sains Malaysia, MALAYSIA

## Abstract

**Introduction:**

The highest number of maternal deaths occur during labour, delivery and the first day after delivery highlighting the critical need for good quality care during this period. Therefore, for the strategies of institutional delivery to be effective, it is essential to understand the factors that influence individual and household factors to utilize skilled birth attendance and institutions for delivery. This study was aimed to assess factors affecting the utilization of institutional delivery service of women in rural districts of Wolaita and Dawro Zones.

**Methods:**

A community based cross-sectional study was done among mothers who gave birth within the past one year preceding the survey in Wolaita and Dawro Zones, from February 01 –April 30, 2015 by using a three stage sampling technique. Initially, 6 districts were selected randomly from the total of 17 eligible districts. Then, 2 kebele from each district was selected randomly cumulating a total of 12 clusters. Finally, study participants were selected from each cluster by using systematic sampling technique. Accordingly, 957 mothers were included in the survey. Data was collected by using a pretested interviewer administered structured questionnaire. The questionnaire was prepared by including socio-demographic variables and variables of maternal health service utilization factors. Data was entered using Epi-data version 1.4.4.0 and exported to SPSS version 20 for analysis. Bivariate and multiple logistic regressions were applied to identify candidate and predictor variables respectively.

**Result:**

Only 38% of study participants delivered the index child at health facility. Husband’s educational status, wealth index, average distance from nearest health facility, wanted pregnancy, agreement to follow post-natal care, problem faced during delivery, birth order, preference of health professional for ante-natal care and maternity care were predictors of institutional delivery.

**Conclusion:**

The use of institutional delivery service is low in the study community. Eventhough antenatal care service is high; nearly two in every three mothers delivered their index child out of health facility. Improving socio-economic status of mothers as well as availing modern health facilities to the nearest locality will have a good impact to improve institutional delivery service utilization. Similarly, education is also a tool to improve awareness of mothers and their husbands for the improvement of health care service utilization.

## Introduction

Maternal mortality is a sensitive indicator of the quality of a health care system in any country. it refers to death of a woman during the pregnancy, delivery or postpartum period [1]. Globally, 287 000 mothers die from complications of pregnancy and childbirth. Sub-Saharan Africa and Southern Asia accounted for 85% of the global burden of maternal deaths [2]. In sub-Saharan Africa, a woman’s risk of dying from treatable or preventable complications of pregnancy and childbirth over the course of her lifetime is 1 in 22, compared to 1 in 7,300 in the developed regions [3] Maternal mortality of Ethiopia is one of the highest figure, accounting for 676 deaths per 100,000 live births in 2012 [4].

The highest number of maternal death occur during labour, delivery and up to 24 hours postpartum (the first day after delivery) highlighting the critical need for good quality care during this period [5, 6]. Evidence from developed countries shows that for the strategies of institutional delivery to be effective, it is essential to understand the factors that influence individual and household factors to utilize skilled birth attendance and institutions for delivery [7, 8]. Maternal mortality and morbidity can be reduced through access to appropriate health care during pregnancy and delivery. However, in sub-Saharan Africa women continue to face limited access to such services [9]. In order to reduce the risk of maternal and infant morbidity and mortality, especially in places where the general socio-economic status is low, access and utilization of the obstetric services is an effective means [10]. Lack of access to appropriate obstetric care, especially during labor, compounds the risk of adverse fetal outcomes such as death or disability [11]. Skilled attendance during labor, delivery and the early post-partum period could reduce an estimated 16–33% of maternal deaths [12–14]. In Ethiopia, 34% of pregnant women received antenatal care from a skilled provider at least once, that is, from a doctor, nurse, or midwife, for their most recent birth. And one woman in every five (19%) made four or more antenatal care visits during the course of pregnancy. However, only 10% of births were delivered by a skilled provider [4]. The most important barrier to access to health services that Ethiopian women mention is taking transport to a facility, lack of money and distance to a health facility [4].

Improving maternal and infant health continues to be a major challenge such that a woman living in sub-Saharan Africa has higher chance of dying during pregnancy or childbirth, as compared to high-income countries [15]. Institutional delivery service utilization of Ethiopian mothers is low. However, study on the factors contributing for safe delivery service utilization was scarce. Therefore, this study aimed to assess factors associated with institutional delivery service utilization among mothers from Wolaita and Dawro Zones, Southern Ethiopia.

## Methodology

### Study area and design

A population based cross-sectional study was done in Southern Ethiopia in two zones, Wolaita and Dawro, from February 01 –March 30, 2015 to assess institutional delivery service utilization. Wolaita and Dawro Zones are among the 13 zones of Southern nations, nationalities and peoples’ region located in southern Ethiopia. According to 2007 national census, these zones (Wolaita and Dawro) have a total population of 1,52,7908 and 492,742 respectively and projected to be 1,838,073 and 592,768 in 2014 respectively. The zones are characterized by high rural population in which 88.3% and 92.9% of Wolaita and Dawro zone inhabitants were rural dwellers respectively [16]. Wolaita zone has 12 districts whereas Dawro zone has 5 districts. Wolaita Sodo, the capital of Wolaita zone is located 330 km south of Addis Ababa, whereas Tarcha, the capital of Dawro zone is located 510 km southwest from Addis Ababa. Wolaita zone is characterized by its dense population whereas Dawro zone is characterized by its difficult topography.

### Study population and sampling

A three stage sampling technique was applied to select study participants from the overall mothers who gave birth in the last 12 months preceding the survey. In the first stage of sampling technique, 6 districts were selected randomly (i.e. 1) Damot Gale, 2) Boloso Sore, 3) Humbo, 4) Ofa, 5) Tocha and 6) Mareka districts). Then, the second stage of sampling technique was employed by randomly selecting two kebeles (the smallest administrative unit in Ethiopia consisting of nearly 5000 total population) from each selected district. Finally, the third stage of sampling technique was accomplished by selecting study participants through systematic sampling technique from their respective kebeles. Accordingly, 957 mothers were included in the survey.

Sample size was calculated using single population formula based on the following assumptions. Using 27% prevalence of institutional delivery service utilization [17], 95% confidence interval, 5% precision, and 10% non response rate and design effect 3, the final sample size was calculated to be 1000.

### Data collection instrument and procedure

Data was collected by using interviewer administered structured questionnaire. The questionnaire was prepared in English language including all relevant variables based on the objectives of the study and translated to local language (Wolaitigna) for Interview. The information included socio-demographic variables and variables of maternal health service utilization factors prepared by reviewing different literatures and guidelines.

### Data management and analysis

Data were coded and entered using Epi-data version 1.4.4.0 and all analyses were done with SPSS version 20. Proportions and means (SD) were used to describe the study population by explanatory variables and place of delivery. Bivariate logistic regression was done to identify the differentials of institutional delivery utilization in the study population. The important predictors of institutional delivery service utilization were determined using multivariable logistic regression model. Stepwise backward procedure was used for modeling by including variables with significant or marginally significant association in the bivariate logistic regression (p-value <0.25). All statistical tests were considered significant at alpha <0.05.

### Quality control

The interviewers were trained prior to data collection. Pretest was done on 50 mothers in the HumboLarenakebele to ensure appropriateness of the study tools and to acquire common understanding on the assessment tools. There was regular supervision of the data collection process by the investigators and supervisors.

### Ethical consideration

Actual data collection was started after Wolaita Sodo University approved the study. Permission from each district was received and verbal consent was obtained from participants since most of them cannot read and write. Their consent was recorded with questionnaire. Participants were informed that they have full rights to participate or not in the study. Furthermore, the objective of study and benefits were clearly communicated. Respondents were also informed that their responses will be kept confidential. In order to keep their response confidential, response of participants was filled by a questionnaire distinguished by code number.

## Result

### Socio-demographic characteristics of study participants

Nine hundred and fifty seven mothers who gave birth within the past 12 months were involved making a response rate of 95.7%. The mean and standard deviation age of study participants was 27.9 and 5.5 years respectively. Majority of them were married, and found in the age category of 21–30 years which accounts for 96% and 63.1% respectively. The mean family size of study participants was 5.6 persons per household. Wolaita and Dawro ethnic groups made up 97.3% of total participants. (Table 1)

**Table 1:** Socio-demographic characteristics of study participants, assessment of institutional delivery service utilization in Wolaita and Dawro zone, 2015.

Variable		Frequency	Percentage
Marital status of mother			
	Single	22	2.3
	Married	905	96.0
	Divorced	12	1.3
	Widowed	4	0.4
Age of mother			
	≤20	104	10.9
	21–30	604	63.1
	≥31	249	26.0
Religion of mother			
	Protestant	712	74.4
	Orthodox	176	18.4
	Catholic	40	4.2
	Others	29	3.0
Mother’s educational status			
	No education	366	38.4
	Grade 1–8	479	50.2
	Grade 9 and above	109	11.4
Mother's occupation			
	Housewife	658	68.8
	Farmer	31	3.2
	Government worker	13	1.4
	Merchant	232	24.2
	Others	23	2.4
Ethnicity of mother			
	Wolaita	675	70.5
	Dawro	256	26.8
	Others	26	2.7
Husband’s educational status			
	No education	264	27.8
	Grade 1–8	491	51.7
	Grade 9 and above	195	20.5
Husband’s occupation			
	Farmer	671	70.1
	Government	69	7.2
	Merchant	123	12.9
	Other	94	9.8
Family size			
	< 5	307	32.3
	5–6	354	37.2
	>6	290	30.5
Average distance from nearest health facility			
	Below 30 min	432	46.0
	30 min and above	507	54.0

### Maternal health service utilization

Majority (i.e. 765 (82.3%)) of participants visited health facility for ANC service at least once, for their index child. Regarding place of ANC visit, most of them (757 ANC attendee mothers) visited health center and health post; whereas only 8 mothers visited hospital. In Ethiopia, it is recommended that all ANC service provisions must be done either at health centers or hospitals except the first visit, in which the service can be provided at health post level. However, 150 participants visited health post for their second visit and above, where comprehensive service could not be obtained.-Concerning the WHO recommendation of focused ANC visit, it is recommended that at least four ANC visits are needed for a pregnant mother till delivery. Nevertheless, in the current study 197 (20.6%) participants visited at least four times. Majority (75.9%) of study participants prefer health center to get a skilled provider followed by health post and hospital. (Table 2)

**Table 2:** Health service utilization of study participants, assessment of institutional delivery service utilization in Wolaita and Dawro zone, 2015.

Variable		Frequency	Percent
Received ANC service (at least once)			
	No	165	17.7
	Yes	765	82.3
Information was provided about pregnancy-related complications			
	No	282	36.2
	Yes	497	63.8
Wanted pregnancy			
	No	259	29.5
	Yes	618	70.5
Had postnatal care			
	No	720	77.0
	Yes	215	23.0
Preference for ANC follow-up			
	health extension	56	6.0
	health professional	196	21.0
	family	655	70.1
	TBA	21	2.2
	Other	6	0.6
Preference for maternity care			
	Health professional	147	15.9
	Relative	682	73.6
	Traditional birth attendant	38	4.1
	Neighbor	48	5.2
	Other	12	1.3
Preferable place to get skilled provider			
	Health center	712	75.9
	Health post	137	14.6
	Hospital	60	6.4
	Prefer no where	29	3.1
Mothers have to plan to deliver in health institution			
	Don't agree	141	14.9
	Agree	808	85.1

Regarding knowledge towards danger signs of pregnancy, the most commonly known danger sign was bleeding, followed by severe headache and severe fatigue in which 561(58.6%), 228(23.8%) and 216(22.6%) of mothers knew the condition as a danger sign of pregnancy, respectively. However, only 3(0.3%), 18(1.9%) and 19(2%) of study participants knew that convulsion, loss of consciousness and difficulty of breathing are considered as a danger sign of pregnancy, respectively.

The most common delivery related health problem was massive vaginal bleeding followed by birth canal laceration and retained placenta accounting for 86(9%), 23(2.4%) and 19(2%) of mothers affected, respectively.However,only10(1.1%)of study participants faced unconsciousness during their last child birth.

**Fig 1:**
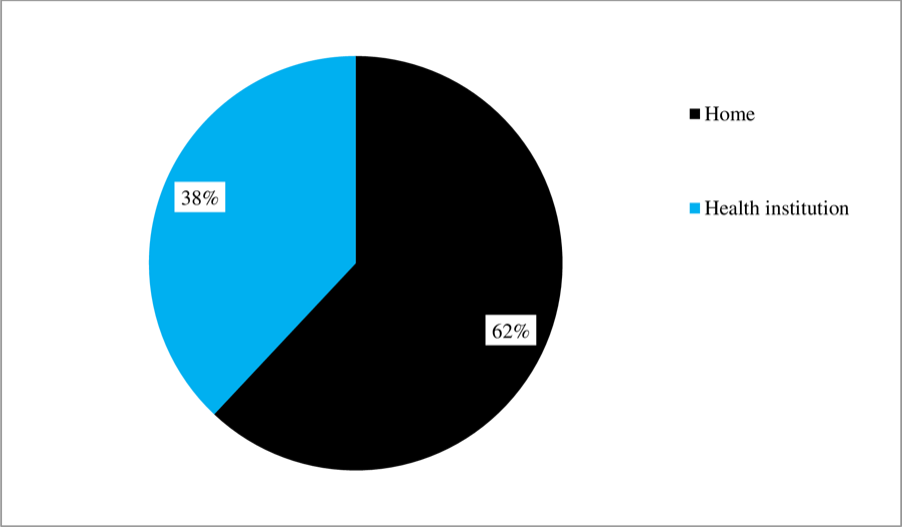
Place of delivery of index child, assessment of institutional delivery service utilization in Wolaita and Dawro zone, 2015.

38% of study participants delivered the index child at health facility (i.e. Health center or hospital), whereas their counterparts delivered outside health facility. However, only 215(23%) of them visited health facility for post natal care. (Table 2 and Fig 1)

Concerning preference for ANC follow up, 655 (70.1%) mothers prefer family members to care for their antenatal care; whereas 196(21%) mothers prefer health professionals. Similarly, 682(73.6%) of mothers prefer family members for maternity care. Seven hundred and twelve (75.9%) of them also prefer health center to get skilled provider followed by health post and hospital, accounting for 134(14.6%), and 60(6.4%), respectively.

### Predictors of institutional delivery

Bivariate analysis indicated that husband’s educational status, age of mother, agreement to give birth at health facility, number of live born children, number of parity, maternal educational status, birth order of index child, wealth index, wanted pregnancy, agreement to visit post natal care, knowledge towards danger signs of pregnancy, distance from nearest health facility, problem faced during delivery, religion and ethnicity of mothers were significantly associated with institutional delivery service utilization.

Factors which showed significant and marginally significant association in Bivariate analysis were further tested by backward stepwise logistic regression, which revealed husband’s educational status, wealth index, average distance from nearest health facility, wanted pregnancy, agreement to follow post natal care, problem faced during delivery, birth order, preference of health professional for ANC and maternity care as the independent predictors of institutional delivery service utilization. (Table 3)

**Table 3:** Predictors of institutional deliveryservice utilization, Wolaita and Dawro Zone, Ethiopia, 2015.

Variable		Inst delivery		COR (95%CI)	AOR (95% C.I)
		Yes	No		
Husband’s educational status					
	No education	75	189		
	primary	185	306	1.52(1.10, 2.11)*	1.53(0.99, 2.38)
	Secondary and above	97	98	2.49(1.69, 3.68)*	2.04(1.20, 3.47) **
Wealth index					
	Lower	82	187		
	Middle	111	158	1.60(1.12, 2.29)	2.01(1.30, 3.09)**
	Higher	100	169	1.35(0.94, 1.93)	1.56(1.01, 2.44)*
Walking distance from nearest health facility					
	≥30 minute	139	368		
	<30 minute	211	221	2.53(1.93, 3.32)	2.21(1.54, 3.17)**
Wanted pregnancy					
	No	72	187		
	Yes	251	367	1.78(1.30, 2.44)**	1.68(1.11, 2.54) *
Agree to follow PNC					
	No	21	107		
	Yes	333	484	3.51(2.15, 5.71)**	1.96(0.95, 4.03)
Problem faced during earlier child birth					
	No	288	510		
	Yes	71	88	1.43(1.01, 2.02)*	1.70(1.06, 2.72) *
Birth order					
	5 and above	83	199		
	3–4	98	186	0.59(0.43, 0.82)**	1.47(0.93, 2.33)
	1–2	167	188	2.13(1.53, 2.96)**	2.10(1.33, 3.30)**
Prefer health professional for ANC					
	No	33	163		
	Yes	326	435	3.70(2.48, 5.53)**	2.69(1.48, 4.88)**
Prefer health professional for maternity care					
	No	22	125		
	Yes	337	473	4.05(2.52, 6.50)**	1.70(1.06, 2.75)*

*- significant at p <0.05

**- significant at p <0.01

## Discussion

In the current study, 38% of study participants delivered their index child at health facility (i.e. Health center or hospital). This shows improvement as compared to the national level of 10% [4] and 27% institutional delivery service utilization prevalence among mothers from Tigray region, Northern Ethiopia [17]. However, the result is nearly in-line with a study done in Uttarakhand, India in which 33% of study participants delivered their index child in health facility [18]. Nevertheless, our finding is far lower than 65.8% institutional delivery service utilization of a study conducted in Kano state, Northern Nigeria [19].

Educational status of head of household is one of the factors which affect institutional delivery service utilization. This study showed that mothers whose husbands were educated for at least secondary level and above were twice more likely to give birth at health facility as compared to mothers whose husbands didn’t attend any formal education, AOR = 2.044(1.206, 3.467). This could be due to the reason that the more a husband (household head) is educated, the more he would be aware of the benefits of institutional delivery, so that he could encourage his wife to give birth at health facility. This is in-line with a study conducted in Woldia and Goba, Ethiopia [20,21].

Household economic status is positively associated with institutional delivery. I.e. mothers who were classified as middle household wealth index were two times more likely to give birth at health facility as compared to mothers from lower household wealth index, AOR = 2.01(1.30, 3.09). Similarly, mothers from higher household wealth index were also 1.6 times more likely to give birth at health facility as compared to mothers from lower household wealth index,AOR = 1.56(1.01, 2.44).This could be due to the fact that eventhough delivery service provision of Ethiopia is free of charge, indirect costs could compromise utilization of institutional delivery. This is in line with a study conducted in Meskan and Mareko districts, Ethiopia [22].

Availability of health facility is also one of the factors which can affect mothers’ utilization of institutional delivery. In the current study, mothers whose residence islocated at a distance of 30 minute or less foot walk from the nearest health facility were two times more likely to give birth at health facility as compared to their counterparts AOR = 2.21(1.54, 3.17).This finding is consistent with a study conducted in Meskan and Mareko districts, Ethiopia, in which distance from health facilities was significantly associated with service utilization (**χ2** = 6.67, p<0.05) [22].

Lack of planning and preparation to have a child could also influence maternal place of delivery. Accordingly, mothers whose index child was a wanted pregnancy were 1.7 times more likely to give birth at health facility as compared to their counter parts, AOR = 1.68(1.11, 2.54). Preference of health professional also determines maternal utilization of institutional delivery. In the current study, mothers who prefer health professional for anti-natal care follow-up were 2.7 times more likely to give birth at health facility, AOR = 2.69(1.48, 4.88); whereas mothers who prefer health professional for their maternity care were 1.7 times more likely to give birth at health facility, AOR = 1.70(1.06, 2.75).Mothers who faced problem during earlier child birth were 1.7 times more likely to give birth at health facility as compared to their counterparts, AOR = 1.698(1.060, 2.721).

As number of parity increases, the probability to give birth at health facility decreases. Accordingly, mothers who had one to two children were twice more likely to give birth at health facility as compared to mothers who have more than five children, AOR = 2.10(1.33, 3.30). This is in line with a study conducted in Uttarakhand, India which indicated that mothers of higher birth order were less likely to deliver with the attendance of skilled personnel as compared to lower birth order [18].

## Conclusion and Recommendation

Eventhough utilization of institutional delivery service shows improvement as compared to earlier national figure; still it is low among mothers of Wolaita and Dawro zone. ANC service utilization also shows improvement. Nevertheless, nearly two in every three mothers delivered their index child outside health facility. Controlling the effect of other variables, husband’s educational status, wealth index, average distance from nearest health facility, wanted pregnancy, agreement to follow PNC, problem faced during delivery, birth order, and preference of health professional for ANC and maternity care were significant predictors of place of delivery. Therefore, improving socio-economic status of mothers as well as availing modern health facilities to the nearest locality will have a good impact to improve the practice of institutional delivery. Similarly, education is also a tool to improve awareness of mothers as well as their husbands for the improvement of maternal health care service utilization.

## References

[1] Nigeria Demographic and Health Survey 2008. Direct estimates of maternal mortality, Federal Ministry of Health, Abuja. pp237-238

[2] World Health Organization: Trends in Maternal Mortality: 1990 to 2010 Estimates developed by WHO, UNICEF, UNFPA and The World Bank. Geneva; 2012.

[3] United Nations: Millennium Development Goals Report. New York: United Nations; 2008.

[4] Central Statistical Agency [Ethiopia] and ICF International. 2012. Ethiopia Demographic and Health Survey 2011. Addis Ababa EaC, Maryland, USA: Central Statistical Agency and ICF International

[5] Khan KS, Wojdyla D, Say L, Gülmezoglu AM, Van Look PFA. WHO analysis of causes of maternal death: a systematic review. The Lancet 2006; 367(9516): 1066–1074. 10.1016/S0140-6736(06)68397-9 16581405

[6] WHO, UNICEF, UNPF: Maternal mortality in 2000. Geneva: Estimates developed by WHO UNICEF and UNFPA WHO; 2004.

[7] Griffiths P, Stephenson R. Understanding user's perspectives of barriers to maternal health care use in Maharashtra, India. Journal of Biosocial Science 2001; 33(3): 339–359 11446398 10.1017/s002193200100339x

[8] Brouwere VD, Tonglet R, Lerberghe WV. Strategies for reducing maternal mortality in developing countries: what can we learn from the history of the industrialized West? Tropical Medicine & International Health 1998; 3(10): 771–782. 9809910 10.1046/j.1365-3156.1998.00310.x

[9] Essendi H., Mills Samuel,and Fotso J-C, (2011) Barriers to Formal Emergency Obstetric Care Services’ Utilization, J Urban Health;88(Suppl 2): 356–369 10.1007/s11524-010-9481-1 PMC3132235 20700769

[10] OchakoR.,FotsoJ-C, IkamariL., and KhasakhalaA., (2011) Utilization of maternal health services among young women in Kenya: Insights from the Kenya Demographic and Health Survey, 2003 BMC Pregnancy Childbirth; 11: 1. Accessed on 3/3/2013 at: http://www.ncbi.nlm.nih.gov/pmc/articles/PMC3022772/10.1186/1471-2393-11-1 PMC3022772 21214960

[11] LuleE.,RamanaG.N.V., OommanN., EppJ., Huntington D. and RosenJ.E. (2005), Achieving The Millennium Development Goal Of Improving Maternal Health: Determinants, Interventions and Challenges, Accessed on 13/12/2013 at: www.worldbank.org/hnppublications

[12] World Health Organization (WHO). Mother-baby package: implementing safe motherhood in countries, World Health Organization, Geneva, 1994.

[13] Magadi M, I. Diamond and R. Rodrigues, Choice of Delivery Care in Kenya, University of Southampton. Available at http://www.socstats.soton.ac.uk Accessed on December 2, 2005.

[14] Bolam A, Manadhar DS, Shestha P, Ellis M. Malla K and Costello AM. Factor affecting home delivery in the Kathmandu Valley, Nepal. Health policy and planning 1998; 13 (2): 152–158. 10180403 10.1093/heapol/13.2.152

[15] Zere E, Oluwole D, Kirigia JM, Mwikisa CN, Mbeeli T. (2011) Inequities in skilled attendance at birth in Namibia: a decomposition analysis. BMC Pregnancy Childbirth. 14; 11:34 doi: 10.1186/1471-2393-11-34 21569585 10.1186/1471-2393-11-34 PMC3118957

[16] Summary and statistical report of the 2007 population and housing census, Federal democratic republic of Ethiopia, 2008

[17] Yohannes A Melaku, Berhe W, Fisaha H T, Semaw F A, Loko A, Alemseged A, et al: Poor linkages in maternal health care services? Evidence on antenatal care and institutional delivery from a community-based longitudinal study in Tigray region, Ethiopia. BMC Pregnancy and Childbirth 2014 14:418 doi: 10.1186/s12884-014-0418-7 25524400 10.1186/s12884-014-0418-7 PMC4279812

[18] Digambar A, Chimankar andHarihar S. Factors influencing the Utilization of Maternal Health Care Services in Uttarakhand. 2011 Ethno Med, 5(3): 209–216

[19] YAR’ZEVER I.S. and Y SAID I.. Knowledge and barriers in utilization of maternal health care services in Kano State, Northern Nigeria.; European Journal of Biology and Medical Science Research 2013; 1(1): 1–14.

[20] Worku A, Jemal M and Gedefaw A. Institutional delivery service utilization in Woldia, Ethiopia. Science Journal of Public Health. 2013; 1(1): 18–23.

[21] Daniel B and Desalegn M. Institutional delivery service utilization and associated factors among child bearing age women in GobaWoreda, Ethiopia. Journal of Gynecology and Obstetrics. 2014; 2(4): 63–70.

[22] Jemal A, Damen H. Determinants of equity in utilization of maternal health services in Butajira, Southern Ethiopia. Ethiopian Journal of Health development. 2012; 26 (Special Issue): 265–270.

